# Supplementary material for: Seasonal Abundance and Diversity of Culicoides Biting Midges in Livestock Sheds in Kanchanaburi Province, Thailand
Source: Insects. 2024 Sep 14;15(9):701. doi: 10.3390/insects15090701 (PMC11432189; doi:10.3390/insects15090701)
Supplement: Supplementary file 1 [file insects-15-00701-s001.zip › insects-3123927-supplementary.pdf]

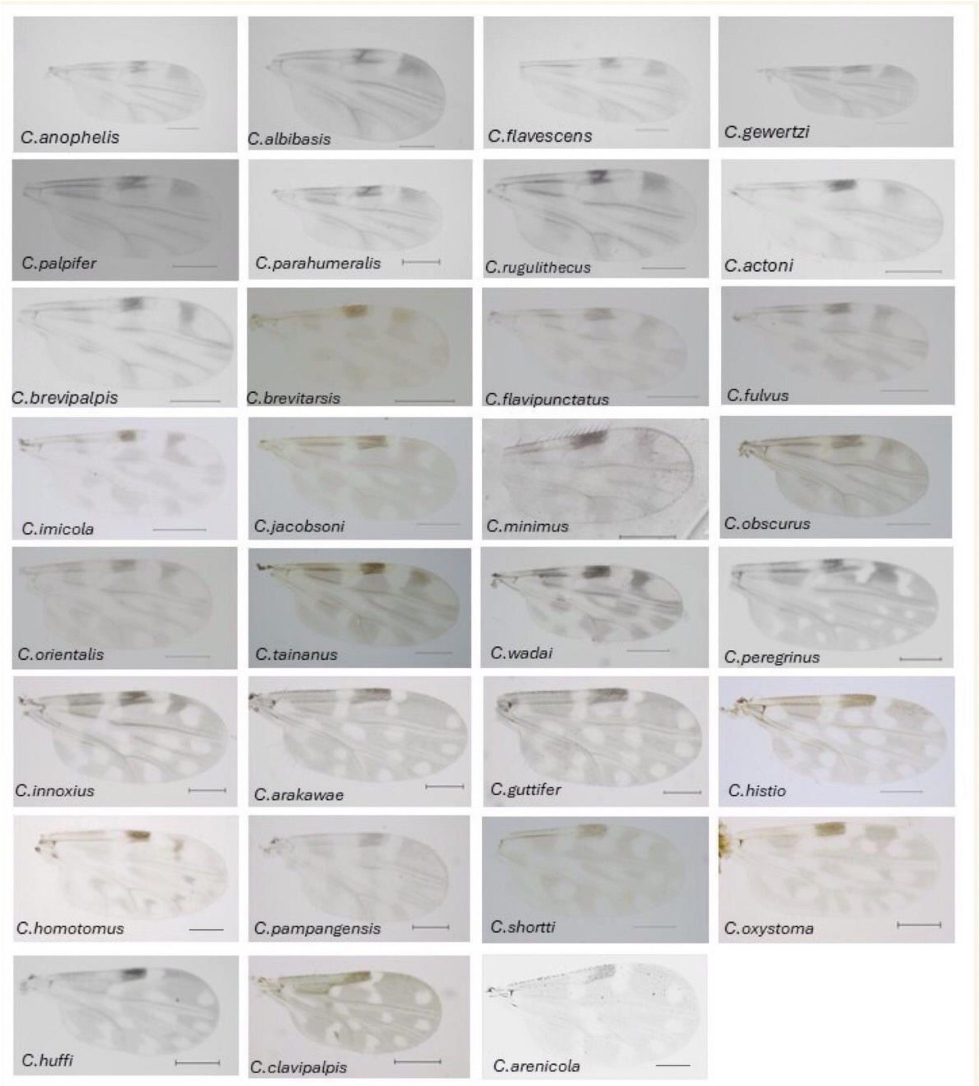

Figure S1. Photos of wing patterns of female *Culicoides* biting midges reported from Tha Maka district, Kanchanaburi province (Bars = 100 μm). These wing patterns were used as a primary key for identifying both male and female of *Culicoides* biting midges. The photos were captured using a stereomicroscope (Olympus, Tokyo, Japan).

Table S1. Distance (m) between two traps calculated from their coordinates (latitude and longitude).

	Trap1	Trap2	Trap3	Trap4	Trap5	Trap6	Trap7	Trap8
Trap2	32.42							
Trap3	68.56	39.33						
Trap4	89.06	94.77	93.09					
Trap5	126.67	122.45	105.30	46.54				
Trap6	158.58	148.30	122.45	85.46	39.33			
Trap7	148.30	144.72	126.67	64.41	22.26	32.42		
Trap8	559.38	544.46	510.65	483.32	437.58	401.56	419.07	
Trap9	570.14	554.24	519.71	495.73	449.69	413.03	431.70	21.61

Table S2 Results of permutational multivariate analysis of variance (PERMANOVA) to determine the effects of date and trap on the community structure of the *Culicoides* assemblage captured by UV-light traps (Tha Maka, Kanchanaburi, Thailand; June 2020 to May 2021).

	**Df**	**Sum of Squares**	R^2	F	**Pr (>F)**
Date	11	6.346	0.213	3.049	< 0.001
Trap	8	6.758	0.227	4.464	< 0.001
Residual	88	6.654	0.560		
Total	107	29.757	1.000		
